# A Contactless Sensor for Pacemaker Pulse Detection: Design Hints and Performance Assessment

**DOI:** 10.3390/s18082715

**Published:** 2018-08-18

**Authors:** Emilio Andreozzi, Gaetano D. Gargiulo, Antonio Fratini, Daniele Esposito, Paolo Bifulco

**Affiliations:** 1 Department of Electrical Engineering and Information Technologies, University of Naples Federico II, Via Claudio, 21-80125 Napoli, Italy; emilio.andreozzi@unina.it (E.A.); daniele.esposito@unina.it (D.E.); 2 Istituti Clinici Scientifici Maugeri S.p.A.—Società benefit, Via S. Maugeri, 4-27100 Pavia, Italy; 3 The MARCS Institute, Western Sydney University, Penrith, NSW 2751, Australia; g.gargiulo@uws.edu.au; 4 School of Life and Health Sciences, Aston University, Birmingham B4 7ET, UK; a.fratini@aston.ac.uk

**Keywords:** pacemaker pulse, coil sensor, pacing monitor, personal healthcare device, pervasive patient monitoring

## Abstract

Continuous monitoring of pacemaker activity can provide valuable information to improve patients’ follow-up. Concise information is stored in some types of pacemakers, whereas ECG can provide more detailed information, but requires electrodes and cannot be used for continuous monitoring. This study highlights the possibility of a continuous monitoring of pacemaker pulses by sensing magnetic field variations due to the current pulses. This can be achieved by means of a sensor coil positioned near the patient’s thorax without any need for physical contact. A simplified model of coil response to pacemaker pulses is presented in this paper, along with circuits suitable for pulse detection. In vitro tests were carried out using real pacemakers immersed in saline solution; experimental data were used to assess the accuracy of the model and to evaluate the sensor performance. It was found that the coil signal amplitude decreases with increasing distance from the pacemaker lead wire. The sensor was able to easily perform pacemaker spike detection up to a distance of 12 cm from the pacemaker leads. The stimulation rate can be measured in real time with high accuracy. Since any electromagnetic pulse triggers the same coil response, EMI may corrupt sensor measurements and thus should be discriminated.

## 1. Introduction

A pacemaker is an active implantable medical device generally used to treat bradyarrhythmias, which are pathologies related to the depression or absence of heart beats. The device is mainly composed by a sensing unit and a pulse generator: the former senses the heart electrical activity and the latter provides electrical stimuli to trigger cardiac contraction when an excessive depression or absence of heart rate is detected.

From 1993 to 2009 in the USA (the largest pacemaker implanter worldwide [1]), the overall use of cardiac pacemakers increased by 55.6% [2]. This positive trend highlights an incessant spread of the pathologies treated with pacemakers and suggests a potential increase in future demand.

Implanted patients must undergo periodic check-ups [3]. Pacemaker follow-up [4] aims to verify the correct operation of the pacemaker to reprogram the device for optimal therapy [5] and to predict the replacement time. Many pathologies treated by pacemaker evolve over time and the continuous adjustment of pacing parameters can improve the efficacy of treatment and offer more benefits to the patient [6,7,8]. However, documentation of patient arrhythmias remains limited by the unreliability of patient symptoms and the need to document the pathology using short- or long-term surface ECG recordings (a Holter device can provide only few days of monitoring with the need to wear electrodes) [9]. For this reason, many pacemakers incorporate some event recorders and counters [4]. As an example, statistics on the occurrence of pacing pulses can be used to measure the evolution of arrhythmias and eventually to tune the electrical or pharmacological therapy. However, not all pacemakers are equipped with event recorders and then provide only concise statistics about pacing, but not information about each pulse timing.

Besides the use of pacemaker programmers, international guidelines [10,11] recommend the use of standard ECG devices to monitor the electrical activity of pacemakers [12,13]. However, precise pacemaker pulse detection and exact timing require high-bandwidth ECG devices [14,15,16].

Accurate pulse detection and timing are used to assess the current battery status [4] at any time without the need for a programmer device. Indeed, in most situations placing a permanent magnet over a patient’s skin, corresponding to the pacemaker, will default to a fixed pacing mode (known as magnet mode) at a rate determined by the battery status. When the pacemaker is in this operating mode, a measurement of the time interval between the pulses provides an indication of the residual battery energy, allowing a prediction of the life expectancy of the device (see the elective replacement time and the end of life [4,17]).

The management of patients with a pacemaker requires regular medical examinations, during which the device should be interrogated using an appropriate programmer and software, and reprogrammed if necessary [4]. However, due to a lack of specific guidelines on pacemaker follow-up (only general guidelines are provided by some national organizations) the frequency of pacemaker follow-up varies from center to center. Generally, follow-up should be every 6 or 12 months, and during the time interval between two follow-ups no information on the pacemaker’s functioning may be known. Moreover, the patients do not have any feedback on the actual functioning of the device; this is known to have a psychological impact on patients’ life (sometimes with devastating consequences), since they perceive themselves as having a vital dependence on the correct functioning of the device [4].

In light of the above information, a device for long-term, real-time continuous monitoring of pacemaker activity is desirable. Such a device would indeed provide detailed reports of pacemaker stimulation events, offering physicians priceless information to help in assessing the progress of the pathological condition. It would also give patients feedback on the correct functioning of their pacemaker (e.g., reporting the date and time of the last pacemaker event detected), thus contributing to improve their quality of life. Finally, it would be useful for the fast assessment of pacemaker battery status.

In a previous paper [18], some of the authors presented an innovative device based on a new methodology to perform contactless, non-invasive monitoring of pacemaker activity. It was based on the detection of the variations of the magnetic induction field caused by the pacemaker current pulses. They can be precisely sensed outside the patient’s body, since the biological fluids and tissues are essentially transparent to the magnetic field, which can propagate outside without distortions or attenuation [19].

**Figure 1:**
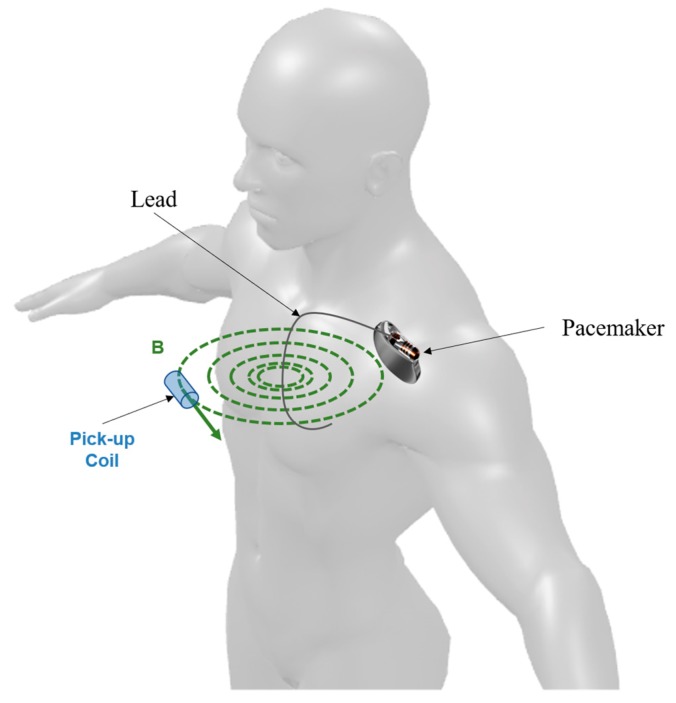
Schematic representation of the sensor operating principle.

The variations of the magnetic induction field are detected by sensing the electromotive force (EMF) induced in a small coil placed close to the patient’s chest, beneath which the pacemaker electrocatheter lies (see Figure 1). Pacemaker pulses are characterized by steep rising and falling edges (generally lasting only a few microseconds), so they cause rapid variations in the magnetic induction field which, in turn, induce an appreciable EMF in the coil (the EMF is proportional to the derivative of the magnetic flux enclosed by the coil).

This method does not require any contact with the patient’s skin, as opposed to those based on electrocardiography. Thus, this sensor allows an easy, contactless, pervasive monitoring of pacemaker pulses.

In this study, a more detailed investigation of the basic principle of the method was carried out. An approximate physical modeling was attempted along with a comparison between the theoretical results and the experimental data, collected by the sensor at increasing distances from the pacemaker lead.

## 2. Materials and Methods

### 2.1. Physical Model

A simplified model of the sensor operating principle was derived. The EMF induced on the pickup coil can be expressed as the sum of the EMFs induced on single loops of the coil:

(1) $$\;\begin{array}{c} EMF=\;\sum_{k=1}^{N}EMF_{k} \end{array}\;$$

The *EMF* induced on the *k*-th loop is related to the magnetic induction field by the Faraday law. Assuming that **B** is constant through the loop surface, it is possible to express the *EMF* as:

(2) $$\;\begin{array}{c} EMF_{k}=\;\oint_{\partial S_{k}}^{}E_{k}\cdot dl=\;-\;\frac{\partial }{\partial t}\iint_{S_{k}}^{}B_{k}\cdot {\hat{n}}_{k}d\Sigma =-\;S_{k}\cos\delta_{k}\frac{\partial \text{B}}{\partial t} \end{array}\;$$

where $S_{k}=S$ is the loop area and $\delta_{k}$ is the angle between the magnetic induction field **B** and the loop surface normal ${\hat{n}}_{k}$.

**Figure 2:**
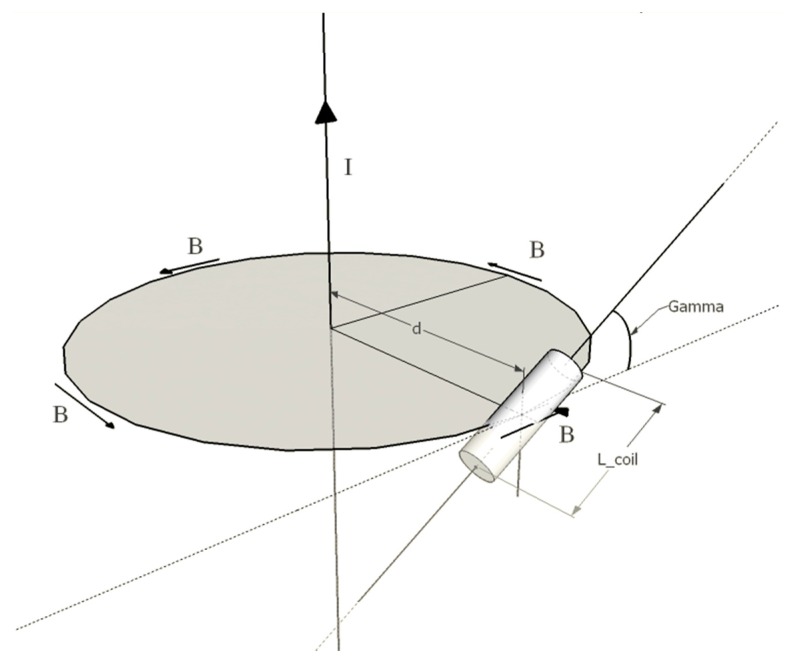
Magnetic coupling of an infinitely long current wire with a coil.

As depicted in Figure 2, the pacemaker lead is assumed to be an infinitely long current wire, thus the magnetic induction field can be derived from the Biot-Savart law. The time derivative of **B** module can be expressed as:

(3) $$\;\begin{array}{c} \frac{\partial \text{B}}{\partial t}=\;\frac{\mu_{0}\mu_{r}}{2\pi d}\frac{dI}{dt} \end{array}\;$$

Since the relative magnetic permeability of biological tissues and fluids is substantially equal to that of vacuum, the magnetic induction field that concatenates with the coil is practically unchanged by patient’s body and the model of the infinitely long current wire in the vacuum can still be used [19]. Furthermore, the vast majority of garments are dielectric and diamagnetic, as both natural fibers (e.g., cotton [20,21]) and synthetic ones (e.g., polyester [22]) do not have an electromagnetic shielding effect. Only very innovative textile materials, which are intentionally made to be conductive [23], can have some effect.

However, this model has some limitations. Indeed, it has to be considered that, in a real pacemaker implant, the lead is not an infinitely long straight wire, but it bends according to the patient’s venous path. Moreover, the ionic currents which flow through the body, closing the circuits between the electrodes (return currents), should be considered as well. These return currents are distributed through the patient’s volume conductor, respecting local differences in tissue resistivities, and their paths are patient-specific and thus very difficult to predict. Obviously, these return currents have an opposite direction with respect to the lead current and thus they generate an opposite contribution to the magnetic induction field concatenated with the coil. For the sake of simplicity, their effect is neglected in the first approximation. However, if the coil is small with respect to the pacemaker implant and it is placed very close to the lead, these simplifying assumptions can still be acceptable.

**Figure 3:**
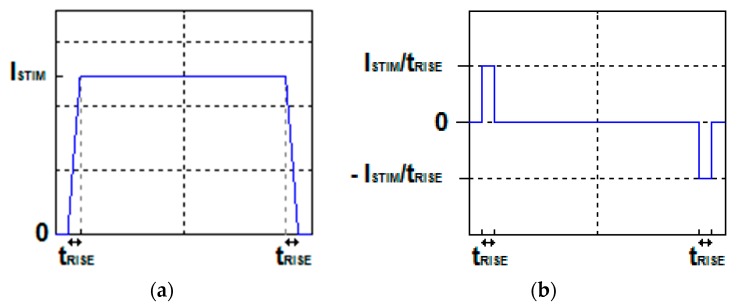
(**a**) Waveform of the stimulation current; (**b**) waveform of the derivative of the stimulation current.

The pacemaker pulse waveform is quite similar to a square wave, so the stimulating current can be assumed to have a square shape of $I_{STIM}$ amplitude, with steep linear rising and falling edges of $t_{RISE}$ duration (Figure 3a). Therefore, the derivative of the current has a waveform that can be represented as a pattern of two opposite polarity square pulses of $I_{STIM}/t_{RISE}$ amplitude and $t_{RISE}$ duration, corresponding to the rising and falling edges of the stimulating current waveform (Figure 3b).

The EMF on the *k*th loop can be expressed as:

(4) $$\;\begin{array}{c} EMF_{k}=-\;S\cos\delta_{k}\frac{\mu_{0}\mu_{r}}{2\pi d}\frac{I_{STIM}}{t_{RISE}} \end{array}\;$$

where $I_{STIM}$ can be expressed as:

(5) $$\;\begin{array}{c} I_{STIM}=\;\frac{V_{STIM}}{R_{el}} \end{array}\;$$

and $R_{el}$ is the electrodes impedance.

Considering the coil positioned as depicted in Figure 2, it is possible to decompose $\delta_{k}$ into two contributions:

1. The angle (γ) between the coil axis and the plane of the magnetic field line;
2. The angle ($\alpha_{k}$) between the **B** vector and the coil axis projection onto the plane of the magnetic field line.

The former results from a rotation of the coil around its transverse axis, so it lies in the plane of the coil axial section orthogonal to the plane of the field line; the latter results from a rotation of the **B** vector direction (tangent to the circular magnetic field line) with respect to the ${\hat{n}}_{k}$ direction (coil axis), so it lies in the plane of the field line.

The $\delta_{k}$ angle can be interpreted as the result of two rotations of ${\hat{n}}_{k}$ with respect to **B** in two orthogonal planes, so the $\cos\delta_{k}$ term can be expressed as:

(6) $$\;\begin{array}{c} \cos\delta_{k}=\cos\alpha_{k}\cos\gamma \end{array}\;$$

Therefore, the EMF of the *k*-th coil loop can be expressed as in Equation (7), and the amplitude of the EMF of the coil as in Equation (8):

(7) $$\;\begin{array}{c} EMF_{k}=\;-\;S\frac{\mu_{0}\mu_{r}}{2\pi d}\frac{I_{STIM}}{t_{RISE}}\cos\gamma \cos\alpha_{k} \end{array}\;$$

(8) $$\;\begin{array}{c} \left|EMF\right|=\;\left|-\sum_{k=1}^{N}S\frac{\mu_{0}\mu_{r}}{2\pi d}\frac{I_{STIM}}{t_{RISE}}\cos\gamma \cos\alpha_{k}\right|=\;S\frac{\mu_{0}\mu_{r}}{2\pi d}\frac{I_{STIM}}{t_{RISE}}\left|\cos\gamma \right|\left|\sum_{k=1}^{N}\cos\alpha_{k}\right| \end{array}\;$$

Since γ and $\alpha_{k}$ assume values in the interval $\left[-\frac{\pi }{2};\;\frac{\pi }{2}\right]$, their cosines are always positive, so the absolute value is unnecessary:

(9) $$\;\begin{array}{c} \left|EMF\right|=\;S\frac{\mu_{0}\mu_{r}}{2\pi d}\frac{I_{STIM}}{t_{RISE}}\cos\gamma \sum_{k=1}^{N}\cos\alpha_{k} \end{array}\;$$

An expression of $\cos\alpha_{k}$, which has a functional dependency on the distance *d*, can be found by means of geometric considerations (see Appendix A) and it has been used in the validation of the model with experimental data. However, for the sake of clarity, the term can be approximated with its mean $\bar{\cos\alpha }$, so the amplitude of the EMF can be expressed as in Equation (10), in which contributions of materials, geometry, pulse parameters, and spatial positioning are clearly highlighted:

(10) $$\;\begin{array}{c} \left|EMF\right|=\;\frac{1}{2\pi }\mu_{0}\mu_{r}NS\frac{I_{STIM}}{t_{RISE}}\cos\gamma \bar{\cos\alpha }\frac{1}{d} \end{array}\;$$

**Figure 4:**
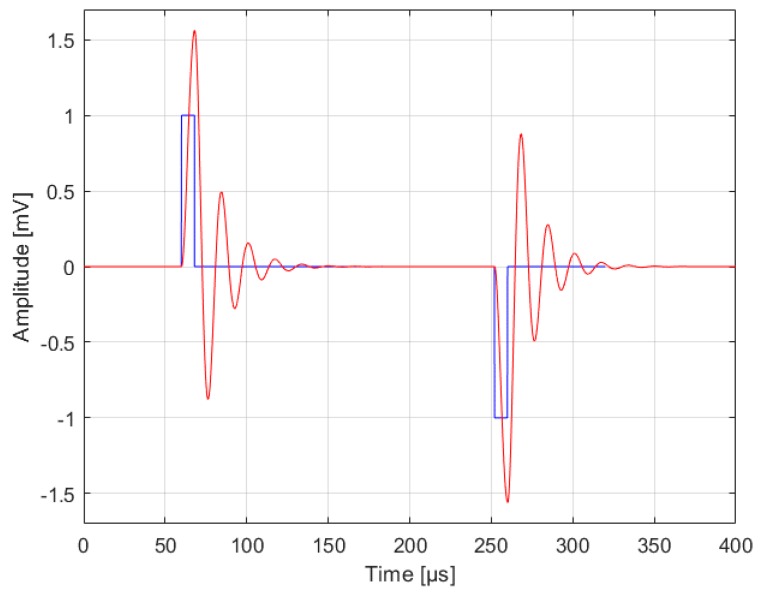
Electromotive force (EMF) pulse (blue line) and coil response (red line) obtained from a simulation of the equivalent RLC circuit model of the coil used for the experimental tests.

Practically, a real coil includes parasitic elements, such as the wire resistance and the stray capacitance between wire wounds. Therefore, a real coil can be modeled as an RLC circuit, showing its self-resonance frequency. So, the actual voltage measurable across the coil would be not the EMF pulse, but the equivalent RLC circuit response to that pulse. Therefore, the peak of the actual coil voltage depends on the EMF pulse duration and the RLC damping ratio (see Figure 4). This must be taken in account when comparing theoretical and experimental results, as the former refer to the EMF (following Equation (10)) and the latter refer to the actual voltage measured across the coil.

### 2.2. Experimental Measurements of Coil Response

**Figure 5:**
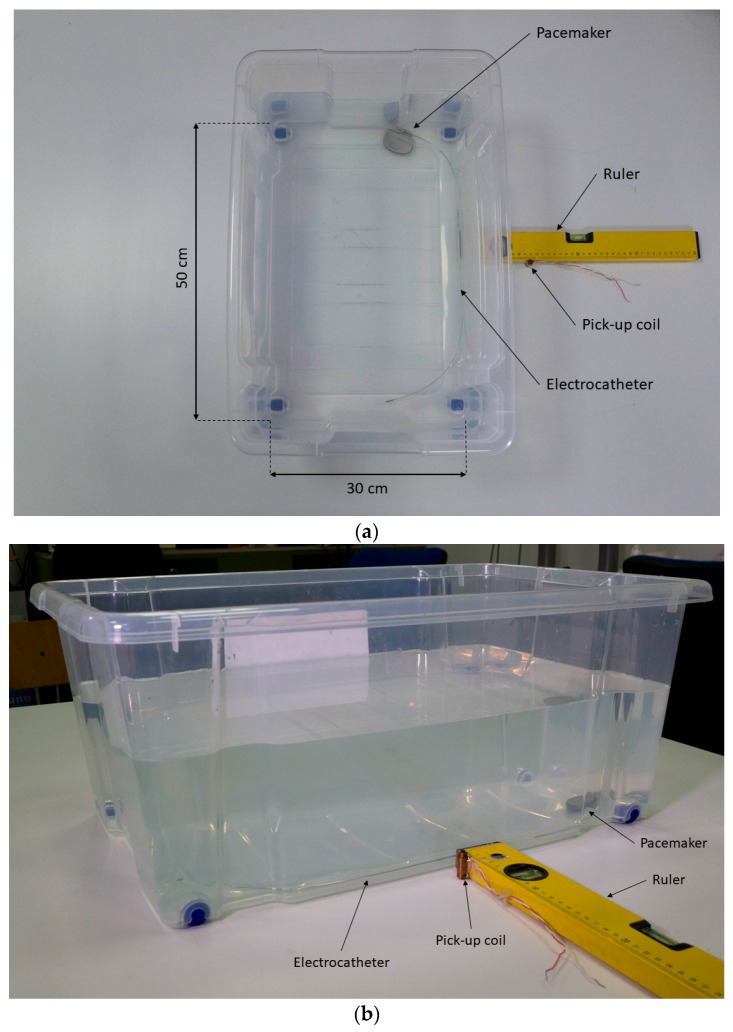
Measurement setup. (**a**) Top view; (**b**) side view.

Various experimental tests were carried out in vitro with a measurement setup (Figure 5a,b) consisting of a torso simulator [24], a St. Jude Medical™ Accent MRI pacemaker, a pickup coil with ferromagnetic core, an amplifier circuit, and a Fluke 123 Industrial Scopemeter oscilloscope. The torso simulator is essentially a plastic box filled with at least 27 L of 0.027 M saline solution to simulate the electrical characteristics of a human torso, which has a mean resistivity of 375 Ω·cm.

The pacemaker was programmed to provide unipolar continuous pacing at a fixed rate of 50 bpm.

**Figure 6:**
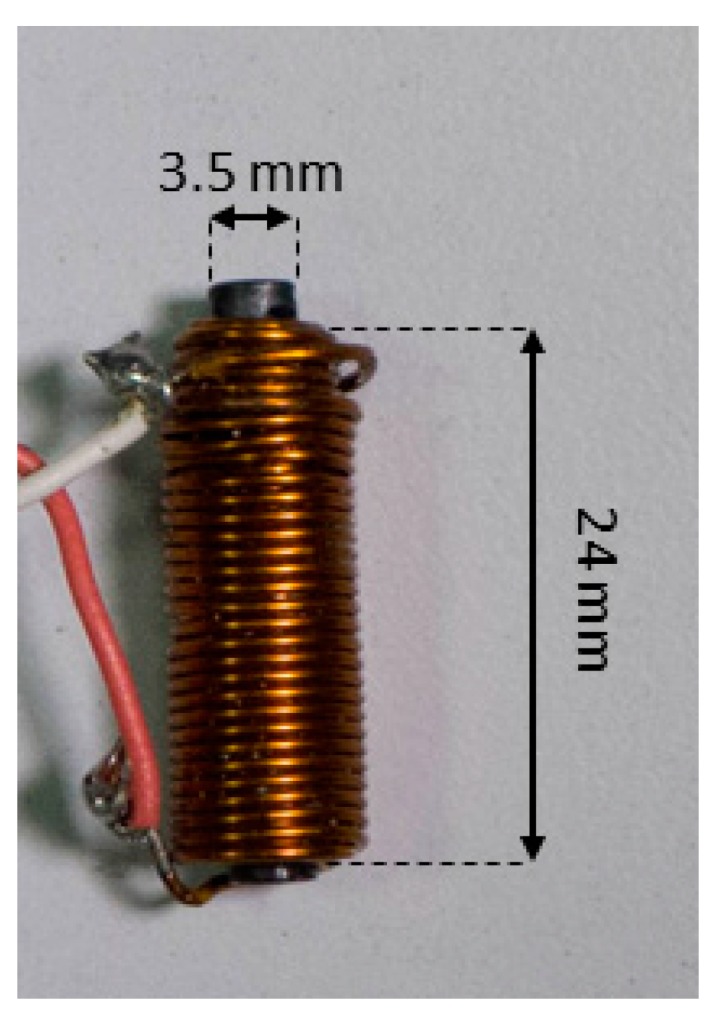
Pick-up coil.

A small coil (Figure 6) with about 55 turns, wound on a 2.4-cm long and 0.35-cm thick cylindric ferromagnetic core, was used to pick up the magnetic induction field generated by the pacemaker current pulses. The coil was found to have a self-inductance of 156 µH and a resistance of 21.91 Ω (measured with a GWINSTEK LCR-816 LCR meter at a frequency of 2 kHz).

**Figure 7:**
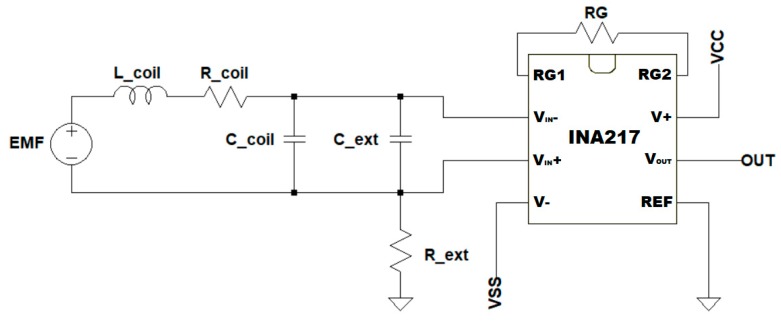
Amplifier circuit based on the INA217 instrumentation amplifier. The input loop is the RLC series circuit model of the coil, with a shunt capacitor to adjust the resonance frequency and a resistor to ground to bias the input of the amplifier. R_G_ is the gain regulation resistor.

Since the voltage across the coil becomes too small as the distance from the pacemaker lead increases, an amplifier circuit was used (Figure 7) based on a Texas Instruments INA217 instrumentation amplifier. The gain was set to 1013 with a gain resistor (RG) of 9.878 Ω to have a sufficiently high amplitude signal. An external shunt capacitor of 42.3 nF (C_ext in Figure 7—measured with the LCR meter at 2 kHz) was added to the input circuit to reduce the natural frequency of the coil. This reduces the input signal bandwidth in order to soften the trade-off on the gain-bandwidth product for the amplifier.

The experimental data were compared to the output produced by a SPICE simulation of the coil equivalent RLC series circuit, forced with two opposite polarity EMF square pulses of 1 mV amplitude and 8 µs duration (as the rising/falling edges of the St. Jude Medical™ Accent MRI pacemaker was 8 µs). The amplitude ratio between the coil response and EMF pulse was then computed.

A validation of the physical model previously derived was also attempted. Actual measurements of the coil signal amplitude were carried out at increasing distances between the coil and the pacemaker lead within a 1–10 cm range, with a 0.25-cm step in the 1–4 cm range, and with a 0.5-cm step in the 4–10 cm range. The experimental data were organized in a distance-to-amplitude curve and then divided by the corresponding $\bar{\cos\alpha }$ factor (computed for every distance value, see Appendix B) in order to obtain a functional dependency only on the distance *d* (refer to Equation (9)).

The experimental results were fitted to the non-linear function defined in Equation (11):

(11) $$\;\begin{array}{c} A=k\;\frac{1}{d} \end{array}\;$$

where A is equal to $\frac{\left|EMF\right|}{\bar{\cos\alpha }}$, d is the distance between the pacemaker lead and the coil, and *k* is a coefficient that groups all of the distance-independent terms in Equation (10), the amplifier gain G, and the amplitude ratio $A_{r}$ between the actual coil response and the coil induced EMF, as reported in Equation (12).

(12) $$\;\begin{array}{c} k=GA_{r}\left[\frac{1}{2\pi }\mu_{0}\mu_{r}NS\frac{I_{STIM}}{t_{RISE}}\cos\gamma \right] \end{array}\;$$

### 2.3. Assembled Sensor Architecture

**Figure 8:**
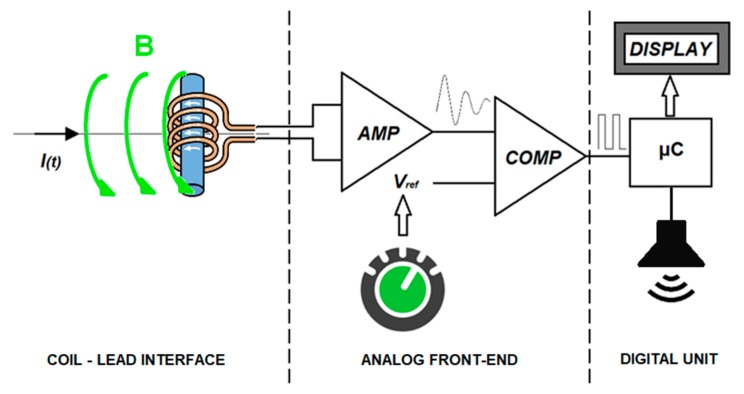
Schematic representation of the architecture proposed for the sensor.

The architecture proposed for the sensor (Figure 8) is mainly composed of two sub-systems: an analog front-end and a digital unit. The first sub-system pre-processes the analog signal picked up by the coil to provide digital signals corresponding to the pacemaker pulses. The second sub-system provides an accurate timing of the pacemaker pulses and the stimulation rate.

#### 2.3.1. Analog Front-End

The front-end was principally designed to amplify the pulses picked up by the coil, in order to obtain a convenient high-amplitude signal, even when the coil is not so close to the pacemaker lead, and to compare them with a variable threshold voltage. In addition, another amplifying circuit was designed to achieve a reduced power consumption and to use a single supply voltage in order to make it suitable for a battery-powered sensor. The instrumentation amplifier used in the circuit reported in Figure 7 provided high linearity for accurate measurement, but for practical use high linearity is not needed because of the non-linear behavior of the comparator (a potential distortion of the signal waveform would not significantly affect the correct detection of pacemaker pulses).

**Figure 9:**
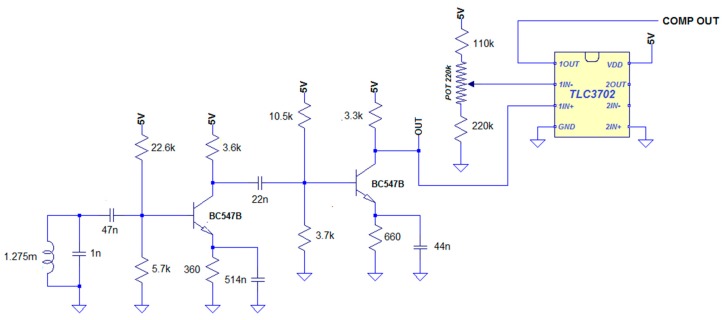
Complete analog front-end schematic: the amplifier circuit feeds the amplified signal to the TLC3702, which compares it with the reference voltage, adjusted by the user with a potentiometer.

The additional amplifying circuit implements a double-stage common emitter amplifier with emitter degeneration (see Figure 9), based on the BC547 bipolar junction transistor, with an overall voltage gain of around 2600. The total current drawn by the circuit, when supplied at 5V, is 2.7 mA.

The analog front-end also encloses the TLC3702 comparator (Figure 9) with a reference voltage adjustable by the user through a potentiometer, so as to produce square pulses corresponding to the peaks of the amplified coil signal. These pulses are sent to the digital unit to detect the presence of pacemaker pulses.

#### 2.3.2. Digital Unit

The digital unit is based on a microcontroller which uses the pulses produced by the analog front-end to trigger hardware interrupts in order to detect the presence of pacemaker pulses. When they are detected, the microcontroller provides acoustic feedback by means of a buzzer and shows the stimulation rate in beats-per-minute on a display. The stimulation rate was computed from the timings of comparator pulses occurrences, measured by means of a digital counter. It is clear that the accuracy of these measurements depends on the number of bits of the counter register and on the clock frequency.

### 2.4. Assembled Sensor Performances

**Figure 10:**
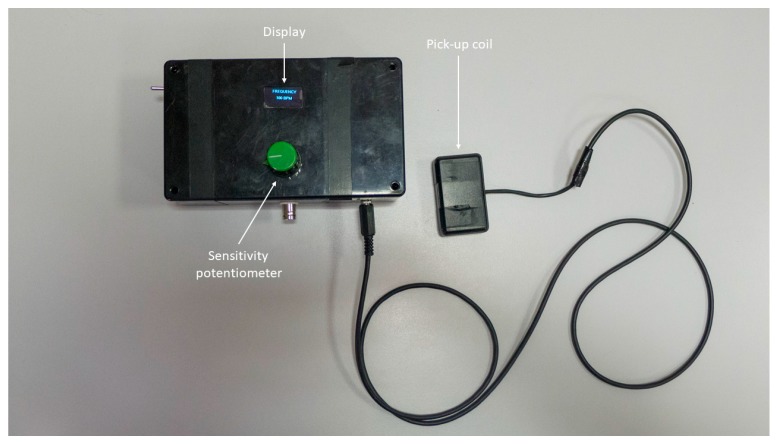
The assembled prototype of the sensor.

The sensor prototype in its actual form is not wearable (see Figure 10), but it can be miniaturized and placed in a small case (the size of a few centimeters) in order to be worn by an implanted patient, e.g., by means of a necklace. The sensor should be placed close to patient’s sternum, either over or under clothing.

The performances of the proposed sensor were assessed by means of in vitro tests conducted with two different pacemaker models, placed inside the torso simulator previously described.

During the tests, in order to verify the capability of the sensor to recognize the pacemaker pulses at increasing distances between the pick-up coil and the pacemaker lead wire, the comparator output signal was monitored to assess the presence of the two short digital pulses generated at the rising and falling edges of the pacemaker pulse. Furthermore, the output of the sensor screen was monitored in order to assess the sensor capability to provide correct information about the pacemaker stimulation rate (expressed in beats-per-minute).

The first tests were carried out with a St. Jude Medical™ Accent MRI, a single-chamber pacemaker, programmed to provide unipolar continuous pacing. Assessment of the stimulation rate measurement was carried out both in continuous pacing (stimulation rate of 50 bpm) and in magnet mode (stimulation rate of 100 bpm [25]).

The second test was carried out with a Boston Scientific Incepta CRT-D, acting as a biventricular pacemaker, programmed to provide bipolar continuous pacing with a stimulation rate of 70 bpm.

## 3. Results

### 3.1. Experimental Measurements of Coil Response

**Figure 11:**
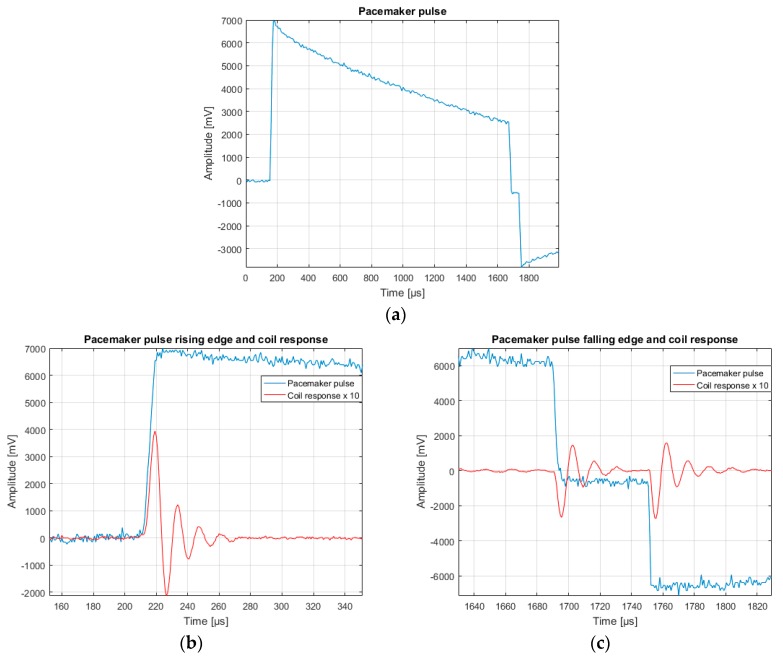
(**a**) Pulse waveform of St. Jude Medical™ Accent MRI pacemaker. It is possible to see a pair of negative falling edges. (**b**) Detail of pulse rising edge and relative coil response. (**c**) Detail of pulse falling edge and relative coil response. As expected, there is a pair of negative damped sine pulses corresponding to the pair of falling edges, i.e., two EMF negative pulses.

Figure 11 shows a simultaneous recording of the pacemaker pulse and the coil output by means of the DSO (Fluke 123 Industrial Scopemeter 20 MHz). The pacemaker pulse (blue line) was recorded using two electrodes immersed in the saline solution (torso simulator), while the coil signal (red line) was acquired at 1 cm from the pacemaker lead and amplified with a gain of 1013.

**Figure 12:**
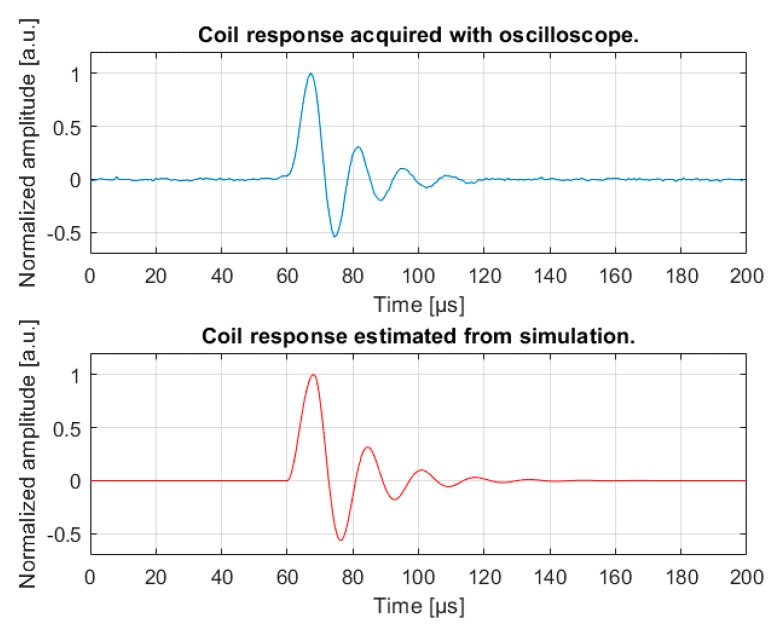
In the upper panel (blue line), the coil response acquired during the tests is shown. In the lower panel (red line), the coil response computed with the simulation is shown.

Figure 12 shows a comparison between the experimental and the simulated coil responses. The simulation of the coil equivalent circuit forced by rectangular EMF pulses of 1 mV with 8 µs rising/falling edges reported an amplitude ratio between the coil response and EMF equal to 1.561 (see Figure 4).

However, the simulated response showed a little lower natural frequency as compared to the acquired response. This is probably due to errors in estimating the coil parameters, which were measured at 2 kHz (the upper limit of the LCR meter).

**Figure 13:**
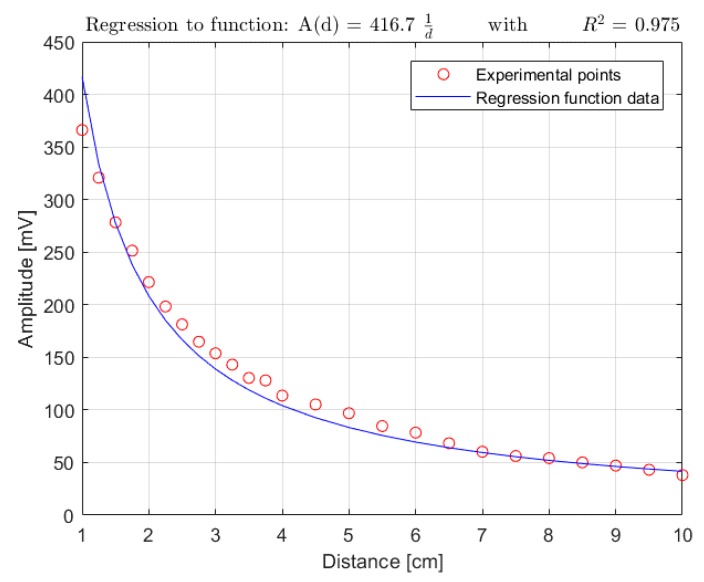
Comparison between experimental points and regression function curve.

The *k* value, computed using Equation (12) with the model parameters reported in Table 1, was found to be 3870 mV·cm. The best-fitting function (Figure 13) scored an R^2^ of 0.975, but with a fitted *k* value of 416.7 mV·cm. The mismatch between the computed and the fitted values of *k* requires detailed comments, which are presented in Section 4.1.

### 3.2. Assembled Sensor Performances

The sensor was able to easily detect the stimulation pulses up to a distance of 12 cm from the pacemakers leads, and to provide a correct measure of the stimulation rate with both the St. Jude Medical™ Accent MRI and the Boston Scientific Incepta CRT-D. The pacing rate was computed by the microcontroller in real time by measuring the time interval between two consecutive pulses by means of an internal counter. For every detection event a counting error of ±1 count must be considered, so for two consecutive pulses the absolute maximum error (in the worst case) is equal to 2 counts. The use of a 16-bit counter at 16 MHz clock frequency, pre-scaled by 1024, guaranteed an accuracy better than 0.0256%, which was obtained for a high pacing rate (120 bpm).

However, it was observed that intense electromagnetic interference sources, such as switching power supplies, digital screens, wireless devices (e.g., mouse, keyboard, etc.) are able to induce EMF pulses with sufficiently high amplitude to be detected from the sensor at short distances, thus corrupting the measurements of the stimulation rate.

## 4. Discussion

### 4.1. Physical Model Validation

The regression presented in Figure 13 reported an R^2^ of 0.975 with a fitted value of the *k* parameter considerably smaller than that analytically derived. This discrepancy can be ascribed to the oversimplification of the proposed model neglecting the ionic return currents, which close the electrical loop inside the saline solution of the torso simulator and determine negative contributions to the magnetic induction field concatenated with the pick-up coil, as they are opposite in direction to the electric current of the pacemaker lead, thus reducing the amplitude of the overall EMF across the coil.

From a design perspective, the overestimation of the *k* parameter given by Equation (12) would lead to an expected coil voltage amplitude much higher than that actually observed. This discrepancy could easily be compensated by increasing the voltage gain of the amplifier, in order to obtain the expected coil voltage amplitude. In fact, in our work we implemented a similar solution by providing the possibility to manually adjust the comparator threshold while keeping the amplifier gain constant, thus making it possible to detect pulses with variable amplitudes. Each pacemaker pulse has its own peculiar waveform (a sort of “signature” of the implanted device), which corresponds to a specific sequence of EMF pulses picked up by the coil. The threshold must be chosen by considering the lowest amplitude pulse, also considering the attenuation of the received signal depending on the coil motion within a given space. In the prototype device, the threshold can be adjusted by the user, but it may be pre-adjusted for a specific patient once the pacemaker waveform is known.

In fact, coil movements (displacements and/or rotations) with respect to the pacemaker lead wire have the effect to change the amplitude of the received signal (as specified by the formulas reported in Equations (7)–(10)). However, since the thresholding operation is non-linear, it can determine an insensitivity to certain signal amplitude changes. So, if the threshold is set low enough to be overcome by the EMF pulses with the lowest amplitude, but still above the noise level, moderate amplitude changes due to the relative motion of the coil will not affect the pulse detection.

The proposed model highlights the key role played by the $\frac{dI}{dt}$ term in the possibility to detect the pacemaker pulse by means of the coil, as well as the dependencies of the coil signal amplitude on the physical and geometrical parameters of the pick-up coil. However, it cannot provide an exact explanation for the experimental data because of its oversimplifying approximations. Detailed modeling of all physical phenomena which explain the experimental data is beyond the scope of this paper, and would probably be useless for the design of this kind of sensor. In fact, in the human body, the inhomogeneities of the electrical properties of biological tissues, in which the electric field generated by the pacemaker propagates, would make it extremely difficult to compute all of the contributions to the magnetic induction field produced by the ionic return currents. That makes a design methodology based on these computations practically unfeasible and above all disadvantageous, as a simple gain compensation (or threshold adjustment) can provide a more than acceptable correction for the predictive errors in Equation (12).

### 4.2. Assembled Sensor Performances

The sensor was able to easily detect pacemaker pulses up to a distance of 12 cm from the pacemaker lead and to provide correct measures of the stimulation rates both with the St. Jude Medical™ Accent MRI (unipolar) and the Boston Scientific Incepta CRT-D (bipolar).

It is worth noting that the test involving bipolar stimulation differs from the simplifying hypothesis held for the proposed model. In fact, the anode and the cathode both lie at the tip of the pacemaker lead, so equal and opposite currents flow through the lead wires, theoretically providing an almost null overall contribution of the magnetic induction field concatenated with the pick-up coil. The remaining non-null contribution is that of the ionic currents generated by the two electrodes in the saline solution, which are concentrated around the tip of the pacemaker lead, thus showing a weaker magnetic coupling with the pick-up coil as they are at a greater distance from it. Nevertheless, the sensor was able to detect the pacemaker pulses with bipolar stimulation because the edges of the pacemaker pulses in bipolar stimulation are much faster than those in unipolar.

The sensor presents some intrinsic limitations. In fact, the coil response to any electromagnetic impulse is a damped sine curve with the same natural frequency and damping ratio, and it has been observed that sources of intense electromagnetic interference may corrupt measurements of the stimulation rate by triggering coil responses with sufficiently high amplitude to be detected from the sensor at short distances. It is clear that linear filtering would not be effective, while a pattern recognition approach applied to patterns of pacemaker pulses detected by the sensor could lead to better results. Given a pacemaker model, the pattern of its pulses is known, so every different pattern detected by the sensor can be ascribed to interferences and noise. Moreover, the pacemaker pulse detection using the thresholding is effective but can be improved by using more sophisticated signal processing methods, for example matched filters, which require the exact sequence of coil EMF pulses induced by the pacemaker (i.e., its “signature”) to be continuously correlated with the signal actually picked up by the coil [26]. This solution requires the coil signal to be directly sampled (bypassing the comparator) and to be further digitally processed. This would obviously result in an increase of the microcontroller workload and therefore of its performance requirements. Since EMIs are characterized by periodic waves or periodic bursts, it is highly unlikely that they can emulate a specific pattern of EMF pulses induced by the pacemaker, thus misleading the pattern recognition or the matched filters approaches.

## 5. Conclusions

In the present paper, a detailed investigation was carried out on the basic physical principle of the sensor proposed for contactless monitoring of pacemaker activity.

A simplified model developed assuming the pacemaker lead as an infinite long wire, but neglecting the effects of ionic return currents, was proposed. Additionally, validation of the model was attempted with a set of experimental data collected by means of a torso simulator. The results of this comparison indicated that the oversimplifying assumptions adopted in the model lead to an overestimation of the voltage amplitude across the coil. However, introducing a simple gain compensation in the analog front-end of the sensor is sufficient to obtain the coil voltage amplitude expected by the designer.

The sensor was able to easily detect pacemaker pulses up to a distance of 12 cm from the pacemaker lead and to provide accurate measures of the stimulation rates, according to the results of some in vitro tests shown in the paper. To our knowledge, there is not any evidence of similar approaches and results in the literature. Moreover, the sensor performed well for bipolar stimulations as well.

In the course of the present investigation, it was observed that electromagnetic interferences may corrupt the measurements of the stimulation rate, since they can induce EMF pulses that can be detected from the sensor at certain distances and wrongly correlated to the pacemaker activity. This issue could be addressed with a pattern recognition approach, based on the identification of patterns of coil responses associated with the pacemaker pulse parameters (e.g., pulse duration, stimulation rate).

In conclusion, on the basis of the results collected, it could be imagined that the sensor is suitable for a contactless real-time continuous monitoring of pacemaker pulses and has several potential applications. For each correctly sensed pulse, its occurrence time (timestamp) can be stored in memory. Like other event loggers, the timestamp should include information about the date and the time accurate to a fraction of a millisecond. The timestamp should have a consistent format and comply with standards (e.g., ISO 8601 [27]) to allow easy comparison of different records and tracking progress over time. Stored data can be transmitted to a server containing all of the patient’s information as soon as a wireless connection becomes available. Patient data can be directly accessed by authorized doctors or they can be routinely analyzed by specific software designed to recognize potential changes in pacemaker activity. Automated alerts can be delivered to the doctor, attracting his/her attention to potentially ominous signs. In its actual form the sensor can be used to assess the battery level of a pacemaker by simply measuring its stimulation rate in magnet mode. This would be helpful in a context of emergency medicine, as it would allow health professionals to evaluate the depletion of the pacemaker battery without the need for the pacemaker-specific programmer. Moreover, it can be used to implement long-term remote monitoring of pacemaker activity. Some improvements are still necessary, such as the following:

- the reduction of sensor dimensions and power consumption, in order to actually make it a wearable device with adequate battery life;
- the implementation of a pattern recognition approach to address the issue of the measurements corruption due to electromagnetic interferences, which is fundamental for obtaining reliable information from remote monitoring.

Further developments will also involve:

- the integration of the coil sensor with a heart contraction detector, in order to obtain information about the mechanical activity of the patient’s heart, allowing the identification of other important common malfunctions of pacemakers such as failure-to-capture, failure-to-pace (oversensing) and failure-to-sense (undersensing), thus improving the assessment of their correct operation. This can be achieved, for example, with the integration of wearable Bluetooth sensors [28] which would send the gathered information to the proposed pacemaker activity sensor, or to a third data acquisition system (e.g., smartphone, tablet, or another personal portable device);
- the development of an alarm device to help the patient stay away from electromagnetic interferences, which can affect the pacemaker correct operation, alerting him at a safe distance from interference sources, e.g., by providing an acoustic alarm. This is important since a majority of implanted patients are not aware of all possible interference sources, even after proper education provided by health professionals.

## Figures and Tables

**Table 1:** Values used for the model parameters in the experimental validation.

Parameter	Value
*µ_r_*	155
*N*	56
*S*	0.096 cm^2^
*V_STIM_*	7.5 V
*R_el_*	640 Ω
*t_RISE_*	8 µs
cos γ	1
*G*	1013
*A_r_*	1.561

**Figure A1:**
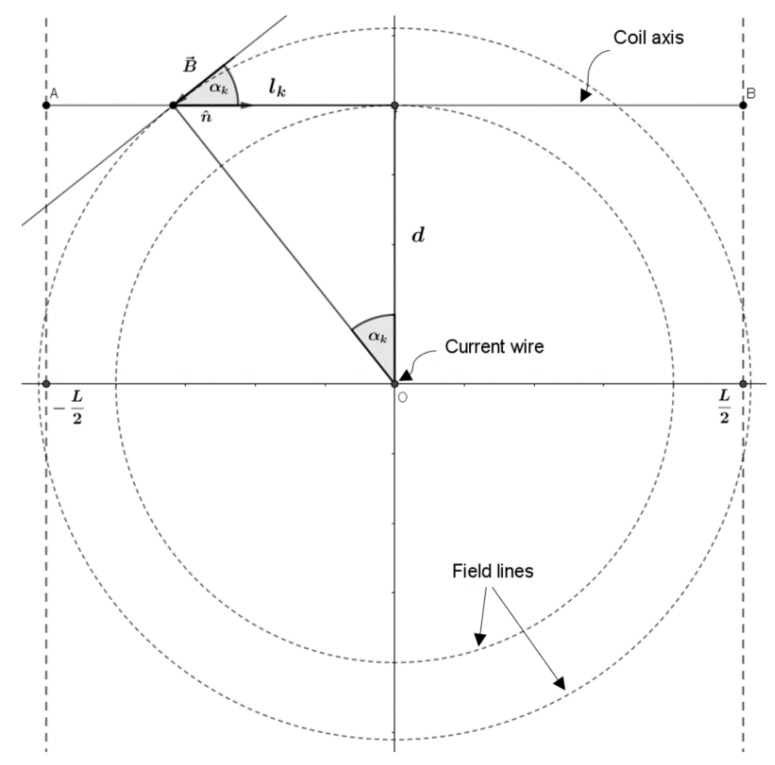
Geometrical representation of the infinitely long current wire with a coil as seen from the top.

## Appendix A

Consider the situation of an indefinitely long current wire with a pick-up coil at distance *d* as seen from the top, with the current wire centered in the origin of a Cartesian reference system (Figure A1).

Let the x axis be parallel to the longitudinal axis of the coil, which is therefore a segment of length *L*, placed at distance *d* from the x axis with its midpoint lying on the y axis.

Consider the circular magnetic induction field line of radius *d*, which is therefore tangent to the segment in its midpoint. Since the normal $\hat{n}$ to all loop surfaces lies on the longitudinal axis of the coil and the **B** vector is always tangent to the circular field line which passes through a generic point, it is clear that, for a loop centered in a generic point P of the coil axis, the angle between $\hat{n}$ and **B** is equal to the angle between the radius of the circular field line passing through P (which is orthogonal to the **B** vector in that point) and the y axis (which is orthogonal to the coil axis, as it is parallel to the x axis).

The factor we want to determine is the $\cos\alpha_{k}$ because it is related to the dot product of the **B** field vector and normal to the generic loop surface. The tangent of this angle can be expressed as:

(A1) $$\;\begin{array}{c} \tan\alpha_{k}=\frac{l_{k}}{d} \end{array}\;$$

where $l_{k}$ is the distance of the center of the *k*th loop from the coil axis midpoint.

The cosine can be expressed as:

(A2) $$\;\begin{array}{c} \cos\alpha_{k}=\;\pm \frac{1}{\sqrt{1+\tan^{2}\alpha_{k}}}=\;\pm \frac{1}{\sqrt{1+\;{\left(\frac{l_{k}}{d}\right)}^{2}}} \end{array}\;$$

Since $\alpha_{k}$ assumes values in the interval $\left[-\frac{\pi }{2};\;\frac{\pi }{2}\right]$, its cosine is always positive, so the ± symbol is unnecessary in practice.

## Appendix B

The analytical computation of the mean value of $\cos\alpha_{k}$, referred to as $\bar{\cos\alpha }$, is presented here. The mean value is defined considering the continuous variable l and the function cosα(l) defined in Equation (A3), and then computing the integral in Equation (A4).

(A3) $$\;\begin{array}{c} \cos\alpha \;\left(l\right)=\;\frac{1}{\sqrt{1+\;{\left(\frac{l}{d}\right)}^{2}}} \end{array}\;$$

(A4) $$\;\begin{array}{c} \bar{\cos\alpha }=\;\frac{1}{L}\overset{\frac{L}{2}}{\underset{-\frac{L}{2}}{\int }}{\left\{1+\;{\left(\frac{l}{d}\right)}^{2}\right\}}^{-\;\frac{1}{2}}dl \end{array}\;$$

Considering the symmetry of the function cosα (l) around 0, the integral can be expressed as in Equation (A5):

(A5) $$\;\begin{array}{c} \bar{\cos\alpha }=\;\frac{1}{L/2}\overset{\frac{L}{2}}{\underset{0}{\int }}{\left\{1+\;{\left(\frac{l}{d}\right)}^{2}\right\}}^{-\frac{1}{2}}dl=\;\;\frac{1}{L/2}{\left[d\;\ln\left(\sqrt{1+\;{\left(\frac{l}{d}\right)}^{2}}+\;\;\frac{l}{d}\right)\right]}_{0}^{\frac{L}{2}} \end{array}\;$$

So, we obtain an expression of $\bar{\cos\alpha }$ as a function of distance d:

(A6) $$\;\begin{array}{c} \bar{\cos\alpha }=\;\frac{2d}{L}\ln\left(\sqrt{1+\;{\left(\frac{L}{2d}\right)}^{2}}+\;\;\frac{L}{2d}\right) \end{array}\;$$
